# Karrikins delay soybean seed germination by mediating abscisic acid and gibberellin biogenesis under shaded conditions

**DOI:** 10.1038/srep22073

**Published:** 2016-02-23

**Authors:** Yongjie Meng, Feng Chen, Haiwei Shuai, Xiaofeng Luo, Jun Ding, Shengwen Tang, Shuanshuan Xu, Jianwei Liu, Weiguo Liu, Junbo Du, Jiang Liu, Feng Yang, Xin Sun, Taiwen Yong, Xiaochun Wang, Yuqi Feng, Kai Shu, Wenyu Yang

**Affiliations:** 1 Key Laboratory of Crop Ecophysiology and Farming System in Southwest China (Ministry of Agriculture), Sichuan Engineering Research Center for Crop Strip Intercropping System, Institute of Ecological Agriculture, Sichuan Agricultural University, Chengdu, 611130, P. R. China; 2 Key laboratory of Analytical Chemistry for Biology and Medicine (Ministry of Education), Department of Chemistry, Wuhan University, Wuhan 430072, P. R. China

## Abstract

Karrikins (KAR) are a class of signal compounds, discovered in wildfire smoke, which affect seed germination. Currently, numerous studies have focused on the model plant Arabidopsis in the KAR research field, rather than on crops. Thus the regulatory mechanisms underlying KAR regulation of crop seed germination are largely unknown. Here, we report that KAR delayed soybean seed germination through enhancing abscisic acid (ABA) biosynthesis, while impairing gibberellin (GA) biogenesis. Interestingly, KAR only retarded soybean seed germination under shaded conditions, rather than under dark and white light conditions, which differs from in Arabidopsis. Phytohormone quantification showed that KAR enhanced ABA biogenesis while impairing GA biosynthesis during the seed imbibition process, and subsequently, the ratio of active GA_4_ to ABA was significantly reduced. Further qRT-PCR analysis showed that the transcription pattern of genes involved in ABA and GA metabolic pathways are consistent with the hormonal measurements. Finally, fluridone, an ABA biogenesis inhibitor, remarkably rescued the delayed-germination phenotype of KAR-treatment; and paclobutrazol, a GA biosynthesis inhibitor, inhibited soybean seed germination. Taken together, these evidences suggest that KAR inhibit soybean seed germination by mediating the ratio between GA and ABA biogenesis.

Soybean (*Glycine max* L.), a legume species native to East Asia, is now widely grown as the primary oilseed crop in the world including in the United States, Brazil, Argentina, India and China^1^,^2^. Although the origin of soybean is debated scientifically, diverse studies have demonstrated that wild soybean forms were present as early as 5000 BC, in China, the Korean Peninsula and Japan^3^. It is noteworthy that China is currently the largest importing country for soybean despite being one of its origins. To meet the increasing demand for food, oil and protein resources, further increases in soybean production are essential.

Timely germination and uniform emergence are key determinants in modern agricultural production systems for many crops, including soybean. Soybean seeds contain high oil and protein contents, and possess a rigid and impermeable seed coat or hull^4^,^5^. These limiting factors result in poor germination and emergence in the field, which significantly decreases soybean yield, especially under stress conditions^6^. Furthermore, soybean seed germination also is a key factor in the food industry, because it remarkably influences diverse nutritional factors in bean sprouts^7^. Therefore, it is worthwhile to investigate the precise molecular and physiological mechanisms underlying the soybean seed germination process.

Few studies have focused on soybean seed germination, compared to the model plants Arabidopsis and rice. A recent study demonstrated that cold plasma treatment significantly promotes soybean seed germination and thereafter seedling establishment and growth processes^4^, although the mechanisms underlying this positive effect are elusive. Furthermore, *GmMFT* (*MOTHER OF FT AND TFL1*) inhibits soybean seed germination^8^, which is similar to the effect of *MFT* in wheat and Arabidopsis^9^,^10^,^11^. However, the precise molecular mechanisms involved in the regulation of soybean seed germination are largely unknown and require further investigation.

Another study showed that hydrogen peroxide (H_2_O_2_) and ethylene possess key roles in this process^12^. After imbibition, H_2_O_2_ production in the seed embryonic axis induces the biogenesis of endogenous ethylene, and then ethylene promotes germination. The promotion effect of H_2_O_2_ has been documented in diverse species, including maize^13^, wheat^14^ and Arabidopsis^15^,^16^, suggesting conserved roles of reactive oxygen species in seed germination in dicots and monocots. Further detailed investigation revealed that H_2_O_2_ down-regulates abscisic acid (ABA) biosynthesis, but up-regulates gibberellin (GA) biogenesis^16^ and, subsequently, the increased ratio between GA and ABA promotes seed germination. Indeed, numerous studies on Arabidopsis demonstrated that ABA and GA play key roles in regulating seed germination, and antagonistically regulate this process^17^,^18^. The contents of ABA and GA are the key determinants during seed germination, and their ratio has pivotal roles^5^,^19^. Therefore, quantification of ABA and GA and analyses of the transcription of key genes involved in hormonal metabolism pathways are validated approaches in the seed germination research field.

Karrikins (KAR) are a small class of signal molecule compounds, discovered in wildfire smoke, which affect seed germination and plant photomorphogenesis in numerous species^20^,^21^. As early as the middle of the last century, scientists found that smoke derived from wild fire could promote seed germination in some species, especially in the diverse Mediterranean-type climate regions including Australia, North America and South Africa^22^,^23^,^24^. However, the molecular structure of KAR remained elusive until 2004, when scientists found that it belonged to the butenolide family, through physiological validation and further Nuclear Magnetic Resonance analysis^20^. So far, six KAR members (KAR_1_ to KAR_6_) have been documented, and are similar in structure to the phytohormones strigolactones (SLs)^25^. Following studies demonstrated that Arabidopsis seeds could respond to KAR treatment, providing a powerful approach to dissect the KAR signaling transduction pathway. Pioneer investigations demonstrated that KAR and SL shared similar signaling transduction pathways^25^,^26^,^27^. In the KAR signaling pathway, KARRIKIN INSENSITIVE 2 (KAI2) perceives the KAR signal and then the interaction between KAI2 and KAR molecules results in the conformational change of KAI2, which activates the MORE AXILLARY BRANCHES (MAX2), a F-BOX protein as E3 ubiquitin ligase; finally, the SCF^MAX2^ complex targets the degradation of repressors, SUPPRESSOR OF MORE AXILLARY GROWTH2 1 (SMAX1) and/or SMAX1-LIKEs (SMXLs)^21^,^25^,^26^,^27^,^28^. The SL signaling cascade has similar performers to the KAR signaling pathway, for example DWARF 14 (D14) is the proposed receptor of SLs and is also a homolog of KAI2; interestingly, MAX2 is a common component in both signaling pathways; furthermore, the repressor DWARF 53 (D53) in the SL signaling cascade shares high sequence similarity with SMAX1 and SMXLs^26^,^27^,^28^. These elegant investigations demonstrated that KAR and SL are evolutionarily conserved, from molecular structure to signaling transduction pathways, and these biological functions indeed overlap, such as in regulating seed germination.

The most attractive function of KAR is the promotion effect for seed germination in some species^20^,^22^. KAR may promote seed germination primarily though enhancing the light response, while the effect of KAR on GA and ABA biogenesis during the seed imbibition process remains largely unknown^29^,^30^. In addition, interestingly, the promotion effect of KAR on seed germination is not detected in dark conditions, suggesting that light is essential for this effect^30^. In contrast to the promotion effect, KAR also shows a negative effect on seed germination in some species: including the arable weeds *Galium aparine*, *Capsella bursa-pastoris*, *Bromus sterilis* and *Alopecurus myosuroides*^31^. These studies demonstrated that KAR possesses distinct biological functions in regard to seed germination, and this effect might depend on plant species genetic background. Importantly, although the primary mechanisms underlying the positive effect of KAR on seed germination were extensively investigated, the mechanisms by which KAR inhibits seed germination remain elusive. Furthermore, the molecular functions of KAR on crop seed germination have not yet been investigated.

Shade stress, with decreased red (R):far red (FR) ratio and light intensity^32^,^33^, results from canopy mixing among neighboring plants especially under the close planting of modern agricultural systems, and significantly reduces crop yield^34^. In the past decade, numerous elegant studies demonstrated that shade affects diverse plant developmental processes including seed germination, branches and tillers, leaf shape, elongation of stems and photosynthesis^32^,^35^,^36^. Interestingly, in Arabidopsis, KAR affects seed germination only under light, rather than under dark conditions, suggested that the effect depends on light. However, whether KAR affects plant seed germination under shaded conditions is largely unknown, especially for crop species.

Here, we report that KAR negatively regulated soybean seed germination and post-germination growth under shaded conditions. In detail, KAR had no effect on soybean seed germination under dark and white light conditions, but had a negative effect under shaded conditions with a concentration-dependent manner. White light had a consistent inhibitory effect on soybean seed germination compared to dark conditions. Further biochemical analysis revealed that KAR positively affected ABA biogenesis but negatively regulated GA biosynthesis during the seed germination process, and the qRT-PCR results for ABA and GA metabolism genes also corresponded with the hormonal quantifications. At the physiological level, the results demonstrated that an ABA biosynthesis inhibitor, fluridone (FL), significantly restored the delayed-germination phenomenon resulting from KAR treatment. Paclobutrazol (PAC), a GA biogenesis inhibitor, showed an inhibitory effect on soybean seed germination, mimicking the KAR treatment. Conclusively, our study revealed that in soybean, KAR had a negative effect on seed germination and post-germination growth through antagonistically affecting ABA and GA biogenesis, which was distinct from the mechanisms in Arabidopsis.

## Results

### KAR negatively regulates soybean seed germination

**Figure 1:**
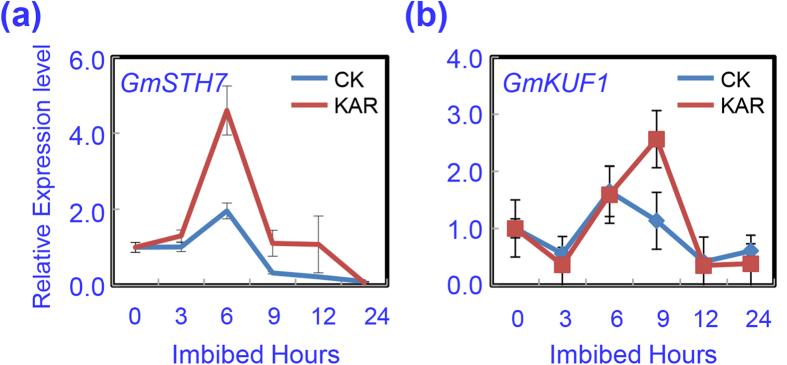
KAR positively regulates the transcription of marker genes during the soybean seed germination process. The expression of KAR-inducible genes (*GmSTH7* and *GmGUF1*) was investigated by qRT-PCR during the imbibition process. Dry seeds and imbibed seeds (3, 6, 9, 12, 24 hours after imbibition) were used for total *mRNA* extraction. Three replications were performed and similar results were obtained. Primer pairs of the marker genes are listed in Supplementary Table 1.

The effect of KAR on soybean seed germination was investigated initially. We firstly confirmed whether KAR treatment in these experiments was valid. Given that a previous study demonstrated that in Arabidopsis, *LIGHT-REGULATED ZINC FINGER 1/SALT TOLERANCE HOMOLOG 3* (*STH7*) and *KAR-UP F-BOX 1* (*KUF1*) were the early response genes for KAR treatment^30^. Therefore, we determined the transcription levels of these genes by qRT-PCR assay after KAR treatment. Soybean seeds were treated by KAR under shaded conditions, and then sampled over a time course. The results showed that *GmSTH7* and *GmKUF1* were significantly up-regulated after KAR treatment in soybean seeds (Fig. 1). These findings demonstrated that the KAR treatment was valid.

**Figure 2:**
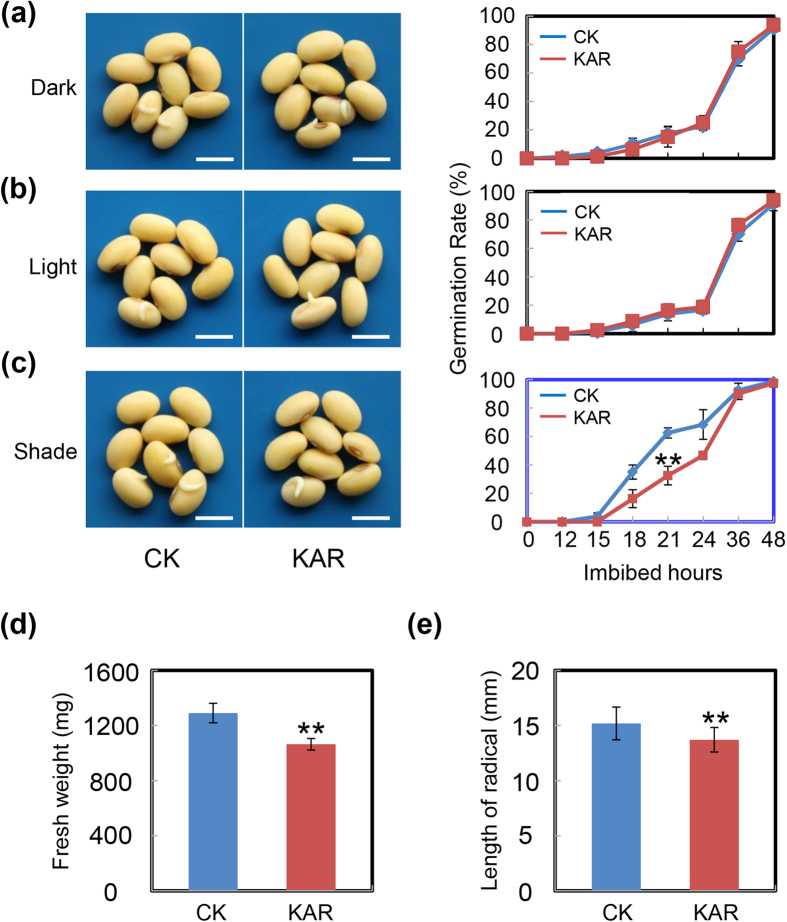
KAR inhibits soybean seed germination under shaded conditions. Healthy soybean seeds (cultivar NN99) were incubated on two layers of filter paper in Petri dishes under dark (**a**), white light (**b**) and shaded conditions (**c**). The concentration of KAR used was 1 μM, and the equivalent ultrapure water was added as control (CK). The germination rates under dark conditions were recorded using a safe green light. Quantitative analysis of germination rates is shown in the right panels. The representative images (21 h after sowing) are shown (left panels). Fresh weight (**d**) and radicle length (**e**) of germinated soybean seeds under shaded conditions were measured. Bar = 10 mm. The average percentages of four repeats ± standard error are shown. **Difference is significant at the 0.01 level.

Former studies demonstrated that KAR affected the seed germination process in a light-dependent manner in Arabidopsis^29^,^30^. Thus several light environments were adopted in the present study. First, using cultivar NN99, we tested the effect of KAR on soybean seed germination under dark conditions. There was no difference in germination rates between KAR-treated and control (CK) seeds over the time course (Fig. 2a), consistent with a pioneer study in Arabidopsis^30^. Next, we performed the same experiments under white light conditions (2340 lx)—to our surprise, we also did not detect any effect of KAR on soybean seed germination (Fig. 2b).

As previous investigations showed that KAR could enhance plant light response^29^,^30^, we speculated that KAR may regulate seed germination under shaded conditions, in which the R:FR ratio and the light intensity is decreased. The experiments performed in the shaded environment revealed that KAR delayed soybean seed germination (Fig. 2c), which was inconsistent with the effect in Arabidopsis^30^, in which KAR promoted seed germination. In detail, the germination rate of KAR-treated soybean seeds was two fold less than that of CK at 21–36 h after imbibition (Fig. 2c). To confirm this, the effect of KAR on soybean post-germination growth under shaded conditions was further investigated. The fresh weight of germinated seeds with KAR treatment was remarkably lower than that of CK seeds (Fig. 2d). Furthermore, KAR also had a significant inhibitory effect on radicle length (Fig. 2e). These results revealed that KAR affected soybean seed germination and post-germination growth processes only under shaded conditions, but not under dark and white light conditions. Altogether, KAR had a negative effect on soybean seed germination, in contrast to investigations with the model plant Arabidopsis.

To further demonstrate the inhibitory effect of KAR on soybean seed germination, we employed another cultivar, ND12, of different genetic origin to NN99. The results also showed that KAR had no effect on soybean seed germination under dark and white light conditions (Supplementary Fig. S1a and b); and the negative effect of KAR on germination was also detected under shaded conditions (Supplementary Fig. S1c). These similar experimental results for different genotypes of soybean cultivars (ND12 and NN99) suggested that KAR indeed inhibited soybean seed germination under shaded, rather than dark and light conditions.

We also investigated whether KAR affected soybean seed germination in concentration-dependent or -independent manners. To this end, treatments with different KAR concentrations were performed. The results showed that, the more obvious inhibition effect was detected when the higher concentrations were employed (Supplementary Fig. S2a). Furthermore, the post-germination growth parameters, including fresh weight (Supplementary Fig. S2b) and radicle length (Supplementary Fig. S2c), confirmed these results. Thus, evidences from these investigations revealed that KAR inhibited soybean seed germination, possibly in a concentration-dependent manner.

### Light delays soybean seed germination

KAR promotes a light response during seed germination^29^,^30^. Because KAR delayed soybean seed germination in the present study, we subsequently speculated that light may negatively regulate soybean seed germination.

**Figure 3:**
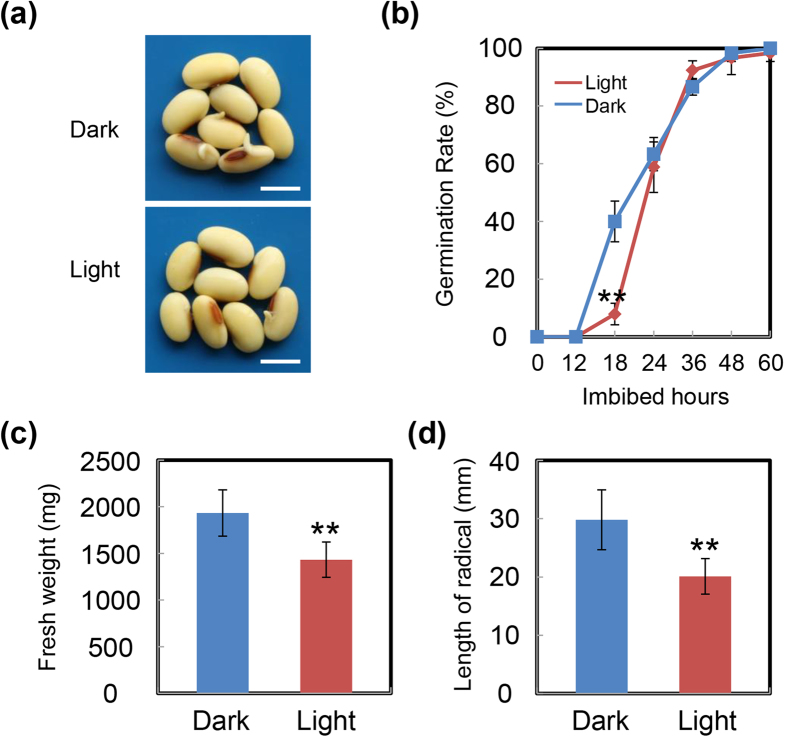
Light inhibits soybean seed germination. (**a,b**) Healthy and uniform soybean seeds (cultivar ND12, yellow soybean) were incubated on two layers of filter paper in Petri dishes at 25 °C under white light (2430 lx) and dark conditions (CK), respectively. The germination rates under dark conditions were recorded using a safe green light. Quantitative analysis of germination rates is shown in the right panels. The representative images (18 h after sowing) are shown (left panels). Fresh weight (**c**) and radicle length (**d**) of germinated seeds were measured. The average length and fresh weight of four repeats are shown. **Difference is significant at the 0.01 level. Bar = 10 mm.

To test this hypothesis, we performed germination experiments under dark and light conditions. We first used the yellow soybean cultivar ND12. Results for germination rates (Fig. 3b), fresh weight (Fig. 3c) and length of radicle (Fig. 3d) of germinated seeds showed that light indeed slowed soybean seed germination. Next, we employed the black soybean variety C-103, of different genetic origin to ND12. Analysis of the diverse parameters including germination rates (Supplementary Fig. S3b), fresh weight (Supplementary Fig. S3c) and length of radicle (Supplementary Fig. S3d) showed that light also delayed C-103 seed germination. These similar results for two different genotypes of soybean cultivars convincingly demonstrated the inhibitory effect of light on soybean seed germination and post-germination growth.

### KAR enhances ABA biogenesis but impairs GA biosynthesis

**Figure 4:**
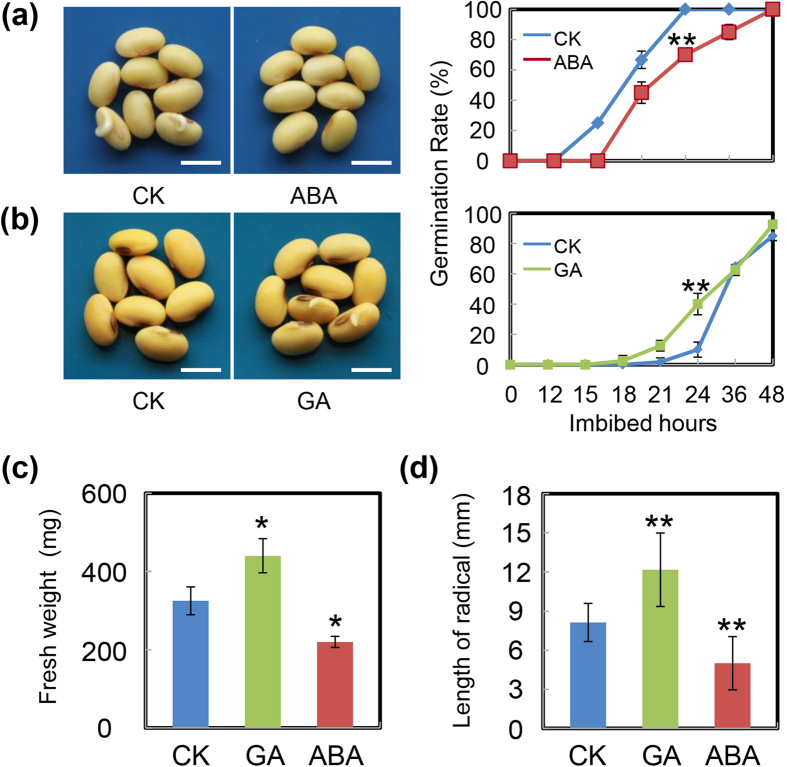
Exogenous ABA inhibits, while GA promotes, soybean seed germination. Soybean seeds (cultivar NN99) were incubated under light (2430 lx) at 25 °C and treated with exogenous 10 μM ABA (**a**) and 10 μM GA (**b**). Equivalent ultrapure water was added as control (CK). Quantitative analysis of germination rates is shown in the right panels. The representative images (24 h after sowing) are shown (left panels). Fresh weight (**c**) and radicle length (**d**) of germinated seeds were measured under ABA treatment, GA treatment and CK. The average length and fresh weight of four repeats are shown. **Difference is significant at the 0.01 level, while *Difference is significant at the 0.05 level. Bar = 10 mm.

Numerous studies have demonstrated that ABA and GA play key roles in the seed germination process. In detail, ABA inhibits while GA promotes seed germination in diverse plant species, including model plants Arabidopsis and rice^17^,^18^,^37^. However, there are no reports concerning the effect of ABA and GA on soybean seed germination. Thus we further examined the influences of ABA and GA on soybean seed germination. The results showed that ABA delayed soybean seed germination (Fig. 4a), while a promotion effect of GA on soybean germination was detected (Fig. 4b). The post-germination growth parameters, including fresh weight (Fig. 4c) and length of radicle (Fig. 4d) of germinated seeds, further confirmed these results. Furthermore, similar to the experiments described above, we also adopted a different genotype of soybean cultivar, ND12, in this experiment. Similar trends of ABA inhibiting and GA promoting soybean germination were documented (Supplementary Fig. S4).

**Figure 5:**
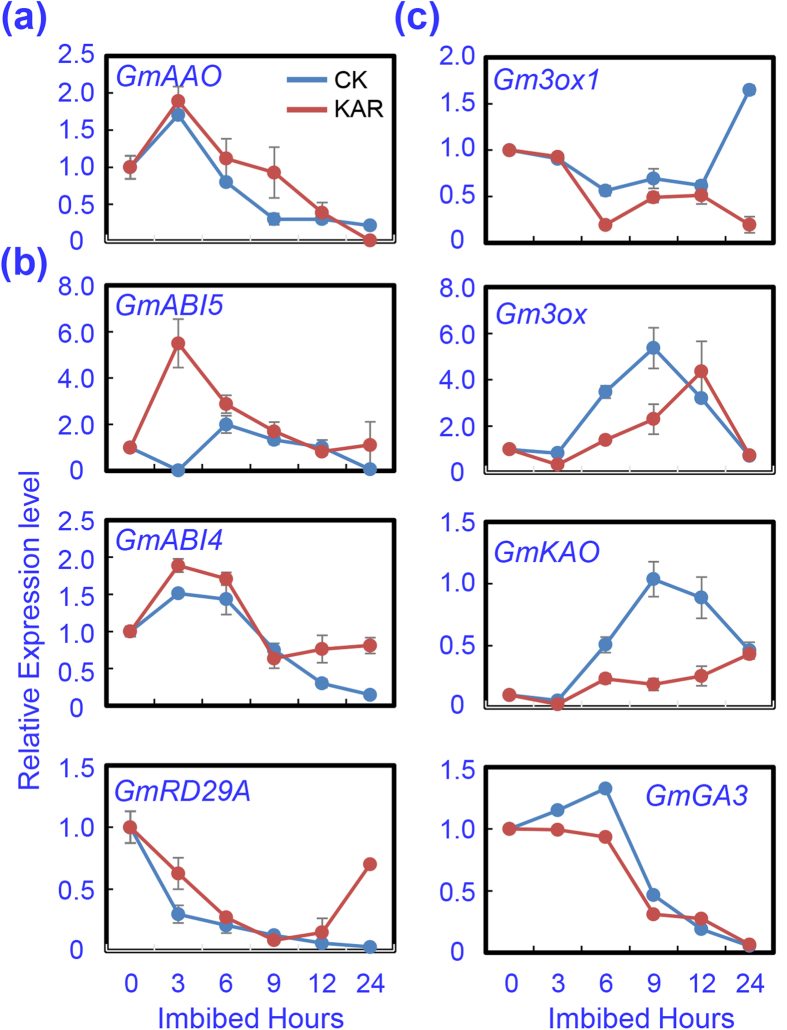
Gene expression analysis during the course of imbibition. Gene expression was investigated by qRT-PCR during the course of imbibition. Dry seeds and imbibed seeds (3, 6, 9, 12, 24 hours after imbibition) were used for total RNA isolation. Three biological replications were performed. (**a**) ABA biosynthesis gene, *GmAAO*; (**b**) positive regulators genes of ABA signaling pathway; and (**c**) GA biosynthesis genes.

To further explore the possible links between the inhibitory effect of KAR on soybean seed germination and GA/ABA biogenesis, we next examined the transcription levels of ABA/GA biogenesis and signaling genes in dry and imbibed soybean seeds with a time-course analysis. The qRT-PCR analysis showed that the transcript level of the ABA biosynthesis gene *GmAAO* significantly increased in KAR-treated seeds after imbibition, compared to the CK (Fig. 5a), especially at 3–6 h after imbibition. Given that the enhanced transcription of the ABA biosynthesis gene may promote ABA signaling and increase the ABA content in seeds, we next investigated the transcription levels of the key genes of the ABA signaling transduction pathway. The results revealed that the expression of *GmABI5* and *GmABI4*, the key positive regulators in the ABA signaling transduction pathway, were remarkably up-regulated after KAR treatment (Fig. 5b). *GmRD29-A*, an ABA-responsive gene, was also significantly induced by KAR treatment during soybean seed germination (Fig. 5b). Altogether, the transcription analysis in regard to the ABA metabolism and signaling genes were consistent with each other, and also demonstrated that KAR may have promoted ABA biogenesis and thereafter signaling.

Previous studies demonstrated that ABA is involved in the suppression of GA biosynthesis during seed germination, and *vice versa*^19^,^38^. Thus we further examined the effect of KAR on the transcription of the key genes involved in the GA biogenesis pathway during soybean seed germination using a qRT-PCR assay. The GA biosynthesis genes, including *GmGA3ox*, *GmGA3ox1*, *GmKAO* and *GmGA3*, were down-regulated by KAR treatment (Fig. 5c). The expression level of *GmGA3ox1* and *GmKAO* in KAR-treated seeds was lower than that in CK, and this trend was maintained during the whole imbibition process (Fig. 5c). In addition, levels of *GmGA3ox* and *GmGA3* were also lower after KAR treatment compared to the CK (Fig. 5c). These analysis results demonstrated that KAR likely negatively regulated GA biogenesis, and thereafter inhibited soybean seed germination.

**Figure 6:**
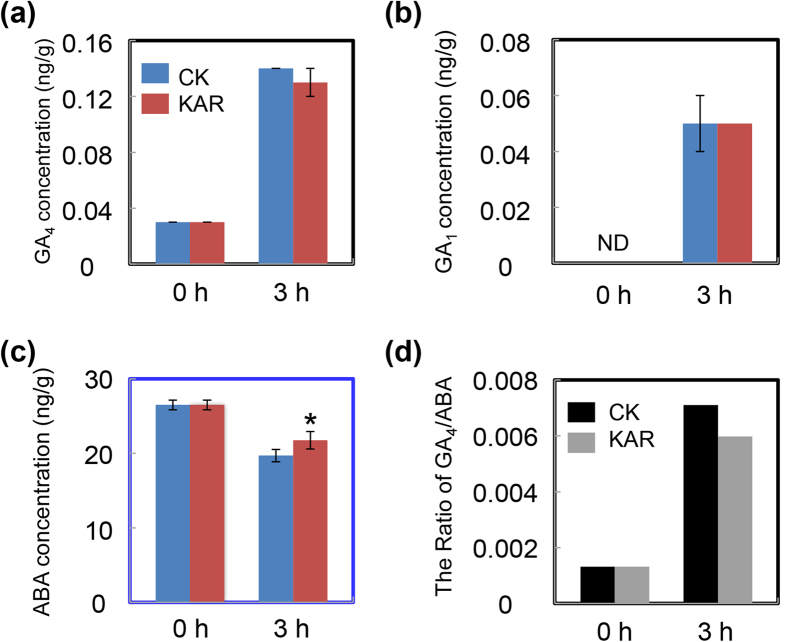
KAR decreases the ratio between GA and ABA. Soybean seeds were incubated under shaded condition at 25 °C and treated with 1 μM KAR. Equivalent ultrapure water was added as control (CK). Dry seeds and 3-h imbibed seeds were used to determine the concentration of endogenous active GA_4_ (**a**), active GA_1_ (**b**) and ABA (**c**). The ratio of GA_4_/ABA (**d**) is also shown. *Difference is significant at the 0.05 level.

The gene expression analysis results described above indicated that the concentrations of ABA and GA might be affected by KAR during the seed germination process (Fig. 5). Consequently, we investigated the endogenous ABA and active GA content in soybean seeds during germination. GA_4_ and GA_1_ are the main active GA structures in plants. The active GA_4_ level in KAR-treated seeds was slightly down-regulated than that in CK, at 3 hours after treatment (Fig. 6a), although the level of GA_1_ did not change in our experiments (Fig. 6b). We further expanded the time-points for GA_4_ quantification, and the results also showed that KAR treatment indeed decreased the GA_4_ level compared to CK, at 3–6 hours after treatment (Supplementary Table S1). This result revealed that KAR negatively affected GA_4_ biogenesis during soybean seed germination. In addition, consistent with the GA_4_ quantification, ABA content in KAR-treated seeds had an increasing trend, compared to CK (Fig. 6c). Importantly, given that the ratio between GA and ABA is the key determinant for seed germination^5^,^19^, we further examined the change of ratio of GA_4_ and ABA after KAR treatment. Consistent with the inhibitory effect of KAR on soybean seed germination (Fig. 2 and Supplementary Figs S1 and S2), the GA_4_/ABA ratio was down-regulated after KAR treatment (Fig. 6d). Consequently, the decreased GA_4_/ABA ratio delayed soybean seed germination as a result of the effect of KAR on transcription of key ABA/GA biogenesis genes.

### FL rescues the delayed-germination phenotype of KAR-treated seeds

The effect of light on soybean seed germination, gene transcription analysis and phytohormone quantifications were consistent with the phenotypic description (Figs 1,3,5 and 6). These investigations demonstrated that KAR delayed soybean seed germination through promoting ABA biogenesis/signaling and impairing GA biosynthesis. To further confirm this conclusion, we tested the responsiveness of soybean seeds to FL, an ABA biosynthesis inhibitor, and PAC, a GA biosynthesis inhibitor.

**Figure 7:**
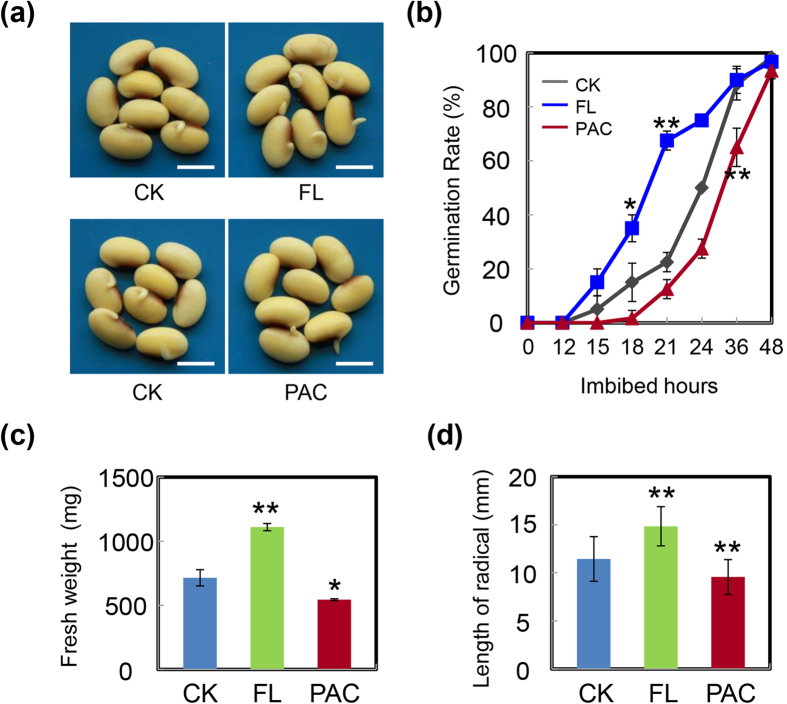
ABA biogenesis inhibitor, FL, and GA biogenesis inhibitor, PAC, affect soybean seed germination oppositely. (**a**) and (**b**) Soybean seeds (cultivar ND12) were incubated on two layers of filter paper in Petri dishes at 250020 °C under light (2430 lx) and treated with 100 nM FL and10 μM PAC. Quantitative analysis of germination rates is shown in the right panels. The representative images (21 h after sowing) are shown (left panels). Fresh weight (**c**) and radicle length (**d**) of germinated seeds were measured under FL treatment, PAC treatment and CK. The average length and fresh weight of four repeats are shown. **Difference is significant at the 0.01 level, while *Difference is significant at the 0.05 level. Bar = 10 mm.

Consistent with the phytohormone measurements, PAC remarkably inhibited soybean seed germination and mimicked the effect of KAR (Fig. 7a,b); the detailed analysis of the fresh weight and length of radicles of germinated seeds also confirmed these results (Fig. 7c,d). However, a promotion effect of FL on soybean seed germination was detected (Fig. 7a,b), and the fresh weight and radicle lengths of germinated seeds provided further supporting evidence (Fig. 7c,d). To demonstrate the effects of FL and PAC on soybean germination, we next employed another soybean cultivar, ND12. Similar to the previous results (Fig. 7), the results for ND12 also showed an inhibitory effect of PAC and an enhancing effect of FL on seed germination (Supplementary Fig. S5).

**Figure 8:**
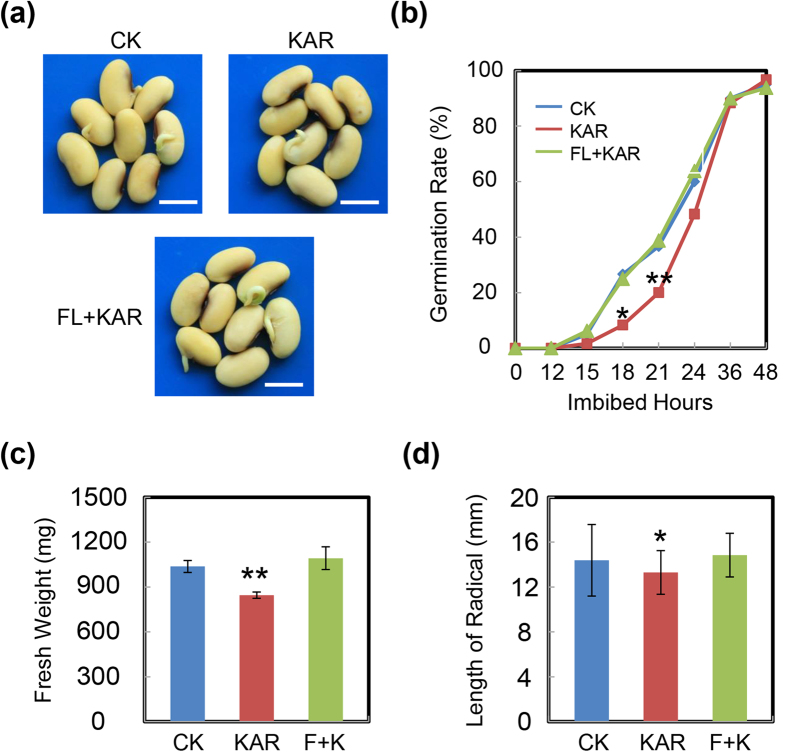
FL rescues the delayed-germination phenotype resulting from KAR treatment. (**a**,**b**) Soybean seeds were incubated on two layers of filter paper in Petri dishes at 25 °C under shade and treated with 1 μM KAR and 100 nM FL + 1 μM KAR, respectively. Quantitative analysis of germination rates is shown in the right panels. The representative images (21 h after sowing) are shown (left panels). Fresh weight (**c**) and radicle length (**d**) of germinated seeds were measured. The average length and fresh weight of four repeats are shown. **Difference is significant at the 0.01 level, while *Difference is significant at the 0.05 level. Bar = 10 mm.

Given that KAR delayed soybean seed germination possibly by enhancing ABA biosynthesis, we further tested the inhibitory effect of KAR in the presence of FL. We speculated that FL could restore the delayed-germination phenotype resulting from the exogenous KAR treatment. Indeed, the results revealed that FL rescued the delayed-germination phenotype of KAR-treated soybean seeds (Fig. 8a,b). Similarly, other phenotypic analyses including fresh weight (Fig. 8c) and radicle length (Fig. 8d) supported these results. Altogether, these investigations demonstrated that KAR indeed promoted ABA biosynthesis during soybean seed germination.

## Discussion

Karrikin was firstly discovered in 2004, and then it was named karrikinolide^39^ and finally karrikin^29^,^40^,^41^. However, the studies in KAR field were concentrated in model plant Arabidopsis, but not in crops. In this study, the detailed physiological analysis of germination, phytohormone measurements and gene expression analysis demonstrated that KAR negatively regulated soybean seed germination and post-germination growth, in a manner distinctly different from that in Arabidopsis. The detailed molecular mechanisms underlying this inhibitory effect were that KAR promoted ABA biogenesis/signaling while impairing GA biosynthesis during soybean seed germination. Altogether, the present investigation demonstrated that KAR not only enhanced seed germination, just like in Arabidopsis, but also might inhibit germination process in other species, just like in soybean.

As Arabidopsis seeds showed responsiveness to KAR treatment, this provides powerful strategies to dissect the actions of KAR, especially the regulatory roles of seed germination and its signaling transduction pathway^21^,^25^. Numerous studies demonstrated that KAR promotes seed germination in a light-dependent manner in Arabidopsis^29^,^30^. It is interesting that, in the present study, KAR negatively regulated seed germination in soybean under shaded conditions, which differs from the conclusions for Arabidopsis.

The data presented in this study strongly demonstrated that KAR delayed soybean seed germination under shaded conditions. First, we performed germination testing in three light environment conditions: dark, white light and shade. Only under shaded conditions was there an inhibitory effect (Fig. 2 and Supplementary Figs S1 and S2). Second, the inhibitory roles of light on soybean seed germination and the action of KAR were consistent. A pioneer investigation showed that KAR enhanced light responses during seed germination^30^. Therefore, if KAR inhibited soybean seed germination, so light should also delay germination. Consistent with this, we indeed detected an inhibitory effect of light on soybean seed germination (Fig. 3 and Supplementary Fig. S3). Taken together, KAR possessed an inhibitory role in soybean seed germination, which differs from investigations in Arabidopsis.

The negative effect of KAR on seed germination was also documented by Daws and colleagues^31^, who found that KAR treatment delayed seed germination of some arable weed species: *Alopecurus myosuroides*, *Bromus sterilis*, *Capsella bursa-pastoris* and *Galium aparine*. This distinct effect of KAR may result from environmental causes. Smoke from combustion of plants stimulates seed germination, especially in fire-following species^20^,^36^. However, in regard to plant species from environments not prone to fire, KAR may have no or even an inhibitory effect on plant growth processes^42^ – a logic following from ecology. It is believed that KAR possesses important but unknown roles during ecological restoration^39^,^43^. Modern soybean cultivars were domesticated by humans for thousands of years, and some weed-related traits or genes were likely lost during evolutionary and/or domestication processes. Wild soybean grows under natural environmental conditions, and thus may be a fire-prone plant. Consequently, in future investigations, it will be interesting to compare the different responses of wild and domesticated soybean cultivars, in regard to the effect of KAR on germination or other physiological processes.

As described in previous studies, KAR may promote seed germination through affecting the GA biogenesis pathway and light responsiveness^29^,^30^. However, whether KAR also regulates ABA biosynthesis and/or signaling pathways remains largely unknown. In the present investigation, we revealed that KAR promoted ABA biosynthesis and thereafter enhanced ABA signaling during soybean seed germination and, finally, delayed the soybean seed germination process.

After confirming the inhibitory effect of KAR on soybean seed germination, we further dissected the underlying molecular mechanisms. The qRT-PCR results revealed that KAR positively regulated some ABA biosynthesis/signaling genes, while negatively affecting GA biogenesis/signaling genes (Fig. 5). Consistent with these data, phytohormone measurements showed that ABA concentration indeed increased with KAR treatment, while GA_4_ content was decreased (Fig. 6a,c). Finally, the GA_4_ and ABA ratio was reduced (Fig. 6d), which delayed soybean seed germination. Furthermore, we found that the GA biogenesis inhibitor PAC also inhibited soybean germination; while the ABA biosynthesis inhibitor FL rescued the delayed-germination phenotype resulting from the KAR treatment (Figs 7 and 8 and Supplementary Fig. S5). These physiological data further demonstrated that KAR inhibited soybean seed germination through respective opposite regulation of ABA and GA biogenesis.

## Methods

### Plant materials and growth conditions

Three soybean (*Glycine max* L.) cultivars with different genetic origins were employed in this study: ND12, NN99 and C-103. In detail, ND12 (yellow soybean) and C-103 (black soybean) are prevailing cultivars in Southwestern China, and were developed by the Nanchong Academy of Agricultural Sciences, Sichuan Province, China. NN99 was screened by the National Center for Soybean Improvement, Nanjing Agricultural University. These three cultivars were grown in the Science & Technology Campus, Sichuan Agricultural University (Chengdu, China), and were harvested at the same time. The elite soybean seeds produced were used for genotypic analysis and further chemical measurements.

### Seed germination phenotypic analysis

Soybean seeds were incubated in 9-cm Petri dishes on two layers of medium-speed qualitative filter papers. A total of 20 seeds were placed in each dish and 12 ml of sterile water was added. Four replicates were performed. The seeds were incubated in a 25 °C incubator (Sanyo Versatile Environmental Test Chamber MLR-350H), with dark, white light or shaded conditions. The light intensity was 2430 lx. Shaded conditions were provided by a covering of green filters, according to a previous protocol^44^. Germination was considered as the detection of the radicle breaking through the seed coat. The germination percentages were calculated and recorded at different time points. Under dark conditions, the germination rates were recorded using a safe green light, according to^45^.

Post-germination growth data including radicle length and fresh weight of germinated seeds were measured 2–3 d after imbibition according to the particular experiment. For each germination test, four experimental replications were performed. The average germination percentage ± SE (standard error) of experiments was calculated.

KAR_2_ possesses the strong activity among the KAR family^46^, and thus was used in this study. KAR_2_ was dissolved in methanol, and then diluted to 1 mM with sterile water as stock. GA and ABA were dissolved in methanol and then diluted with sterile water to 1 and 10 mM as stocks, respectively. Both FL and PAC were dissolved in methanol to 1 mM as stocks. These stocks were diluted to any concentrations as needed in the corresponding experiments. ABA, GA, FL and PAC (product numbers A1049, G7645, 45511 and 33371, respectively) were ordered from Sigma-Aldrich Co. Ltd., USA. KAR_2_ (product number 857054-03-6) was provided by Toronto Research Chemicals Inc., Canada.

### Gene expression analysis

Total RNA preparation (from dry or imbibed seeds at different time points), first-strand cDNA synthesis and the qRT-PCR assay were performed as described by^19^. DNase I-treated total RNA (2 μg) was denatured and then subjected to reverse transcription using Moloney murine leukemia virus reverse transcriptase (200 units per reaction; Promega Corporation). Quantitative PCR was performed using the SsoFast™ EvaGreen Supermix (Bio-Rad). Gene expression was quantified at the logarithmic phase using the expression of the housekeeping *GmACTIN11* RNA as an internal control. Three biological replicates were performed for each experiment. Primer sequences for qRT-PCR are shown in Supplementary Table 1.

### Quantification of ABA in soybean seeds

For analysis of ABA content in dry or imbibed soybean seeds, we used a previous protocol^19^. Seeds were ground in liquid nitrogen, and 300 mg of seed powder was homogenized and extracted for 24 h in methanol containing D6-ABA (OIChemIm Co. Ltd.) as an internal standard. Purification was performed with an Oasis Max solid-phase extract cartridge (Waters) and eluted with 5% formic acid in methanol. Subsequently, the elution was dried and reconstituted, and then injected into a liquid chromatography–tandem mass spectrometry system consisting of an Acquity ultra performance liquid chromatograph (Acquity UPLC; Waters) and a triple quadruple tandem mass spectrometer (Quattro Premier XE; Waters). Three biological replications were performed.

### Quantification of endogenous GA

Endogenous GA was determined as described by^47^. Soybean seeds (400 mg) were frozen in liquid nitrogen, ground to fine powder and extracted with 80% (v/v) methanol. GA isotope standards were added to plant samples before grinding. The crude extracts were purified by reversed-phase solid-phase extraction, ethyl ether extraction and derivatization. The resulting mixture was injected into capillary electrophoresis-mass spectrometry (CE-MS) for quantitative analysis. Three biological replications were performed.

### Statistical analysis

The data including germination rates, fresh weight and radicle length of germinated seeds, and phytohormone quantification results were analyzed using Student’s *t*-test. Image J software was used to measure the length of radicles.

## Additional Information

**How to cite this article**: Meng, Y. *et al.* Karrikins delay soybean seed germination by mediating abscisic acid and gibberellin biogenesis under shaded conditions. *Sci. Rep.* **6**, 22073; doi: 10.1038/srep22073 (2016).

## Supplementary Material

Supplementary Information
