# Supplementary material for: Karrikins delay soybean seed germination by mediating abscisic acid and gibberellin biogenesis under shaded conditions
Source: Sci Rep. 2016 Feb 23;6:22073. doi: 10.1038/srep22073 (PMC4763256; doi:10.1038/srep22073)
Supplement: Supplementary Information [file srep22073-s1.pdf]

# Karrikins delay soybean seed germination by mediating abscisic acid and gibberellin biogenesis under shaded conditions

Yongjie Meng 1, Feng Chen 1, Haiwei Shuai 1, Xiaofeng Luo 1, Jun Ding 2, Shengwen Tang 1, Shuanshuan Xu 1, Jianwei Liu 1, Weiguo Liu 1, Junbo Du 1, Jiang Liu 1, Feng Yang 1, Xin Sun 1, Taiwen Yong 1, Xiaochun Wang 1, Yuqi Feng 2, Kai Shu 1, *, Wenyu Yang 1, *

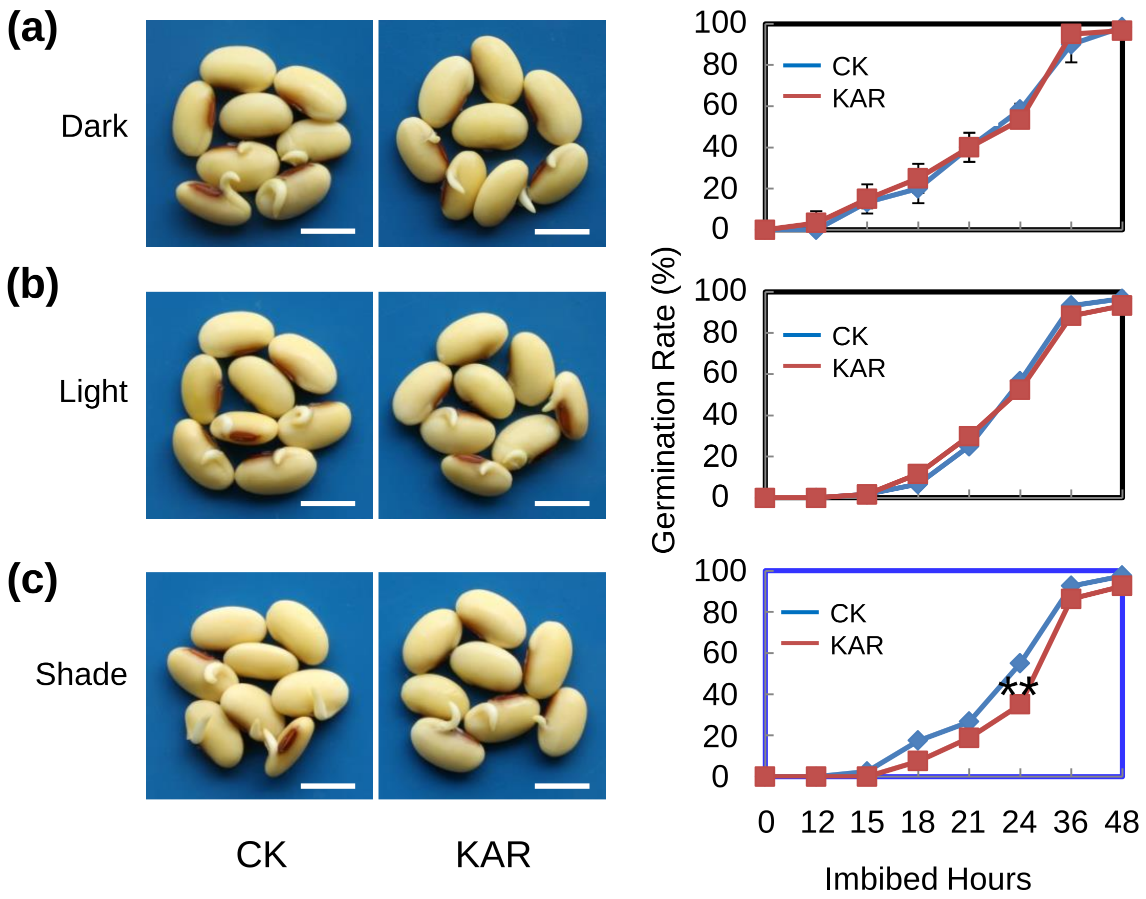


**Supplementary Fig. S1.** KAR inhibits soybean seed germination under shaded conditions. Healthy and elite soybean seeds (cultivar ND12) were incubated on two layers of filter paper in Petri dishes under dark (a), white light (b, 2430 lx) and shaded conditions (c). The KAR concentration used was 1 μM, and the equivalent in ultrapure water was added as control. The germination rates under dark condition were recorded using a safe green light. Quantitative analysis of germination rates is shown in the right panels. The representative images (24 h after sowing) are shown (left panels). Bar = 10 mm. ** Difference is significant at the 0.01 level.

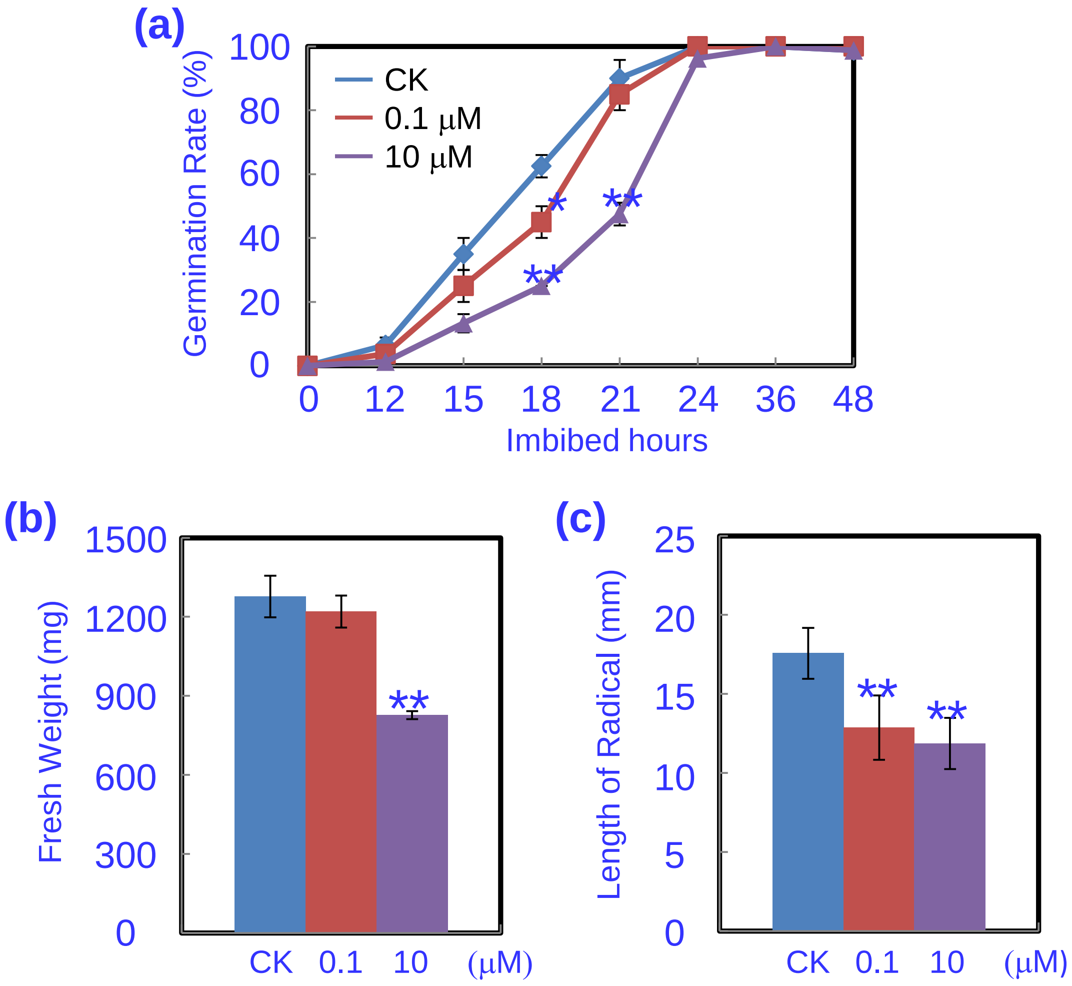


**Supplementary Fig. S2.** KAR inhibits soybean germination in a concentration-dependent manner. Healthy and uniform soybean seeds were treated with different concentrations of KAR and incubated on two layers of filter paper in Petri dishes under shaded light at 25 ° C. Equivalent ultrapure water was added as control (CK). Quantitative analysis of germination rates is shown in (a). Fresh weight (b) and radicle length (c) of germinated seeds were measured. The average percentages of four repeats ± standard error are shown. ** Difference is significant at the 0.01 level, while * Difference is significant at the 0.05 level. Bar = 10 mm.

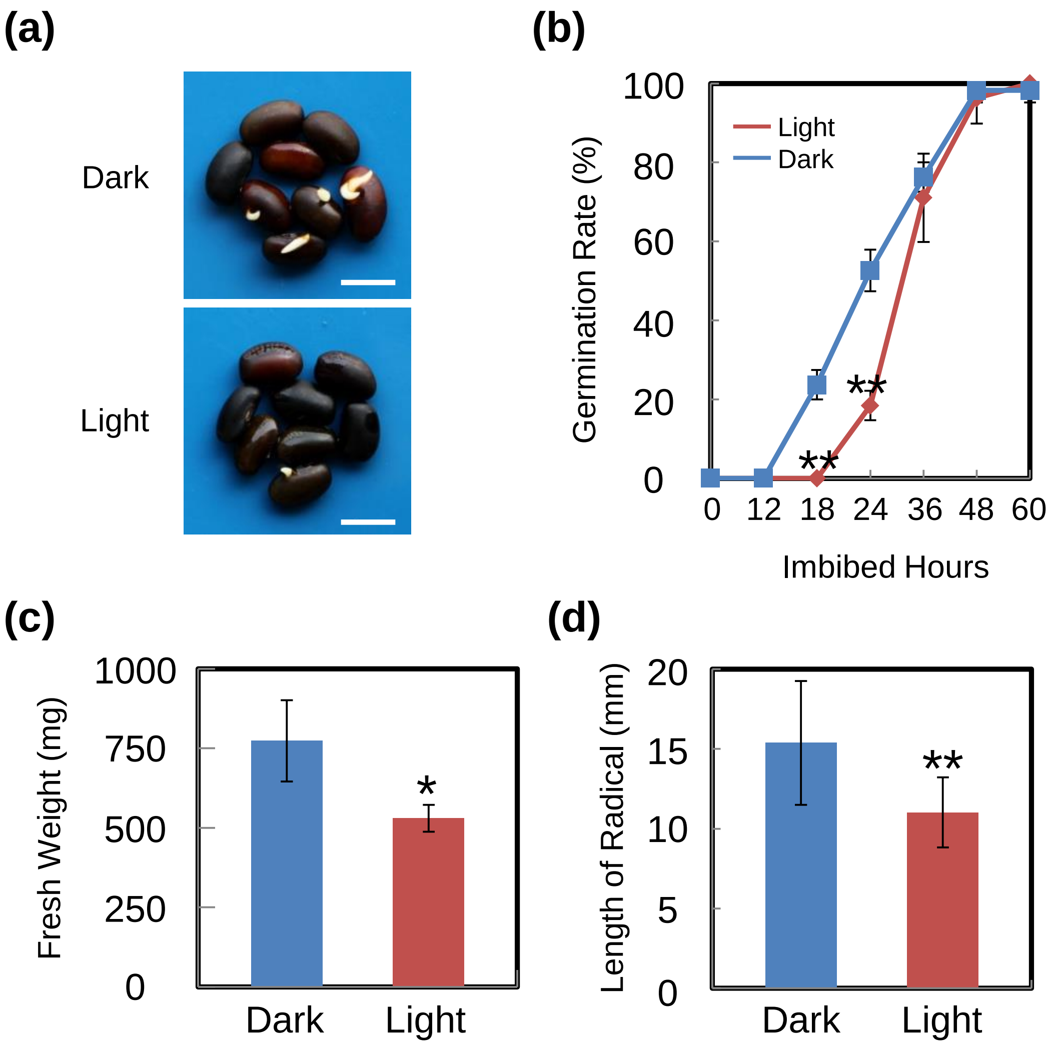


**Supplementary Fig. S3.** Light inhibits soybean germination. (a) and (b) Healthy and uniform soybean seeds (cultivar C-103, black soybean) were incubated on two layers of filter paper in Petri dishes at 25 ° C under white light (2430 lx) and dark conditions (CK), respectively. The germination rates under dark conditions were recorded using a safe green light. Quantitative analysis of germination rates is shown in (b). The representative images (24 h after sowing) are shown (a). Fresh weight (c) and radicle length (d) of germinated seeds were measured. The average length and fresh weight of four repeats are shown. ** Difference is significant at the 0.01 level, while * Difference is significant at the 0.05 level. Bar = 10 mm.

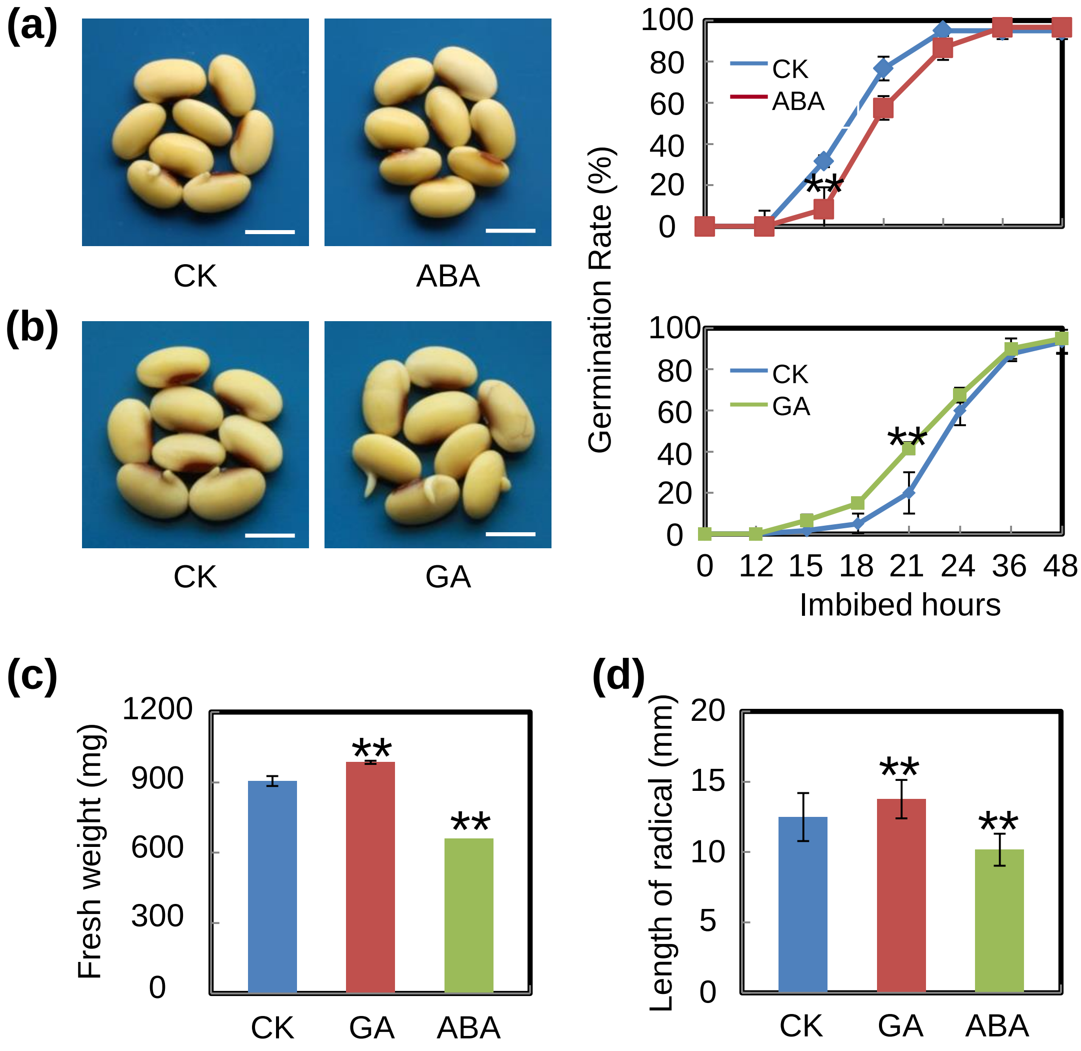


**Supplementary Fig. S4.** Exogenous ABA and GA affect the germination of soybean seeds. Soybean seeds (cultivar ND12) were incubated under light (2430 lx) at 25 ° C and respectively treated with exogenous 10 μM ABA (a) and 10 μM GA (b). Equivalent ultrapure water was added as control (CK). The average percentages of four repeats ± standard error are shown. Quantitative analysis of germination rates is shown in (b). The representative images (21 h after sowing) are shown (a). Fresh weight (c) and radicle length (d) of germinated seeds were measured under ABA treatment, GA treatment and CK. The average length and fresh weight of four repeats are shown. ** Difference is significant at the 0.01 level. Bar = 10 mm.

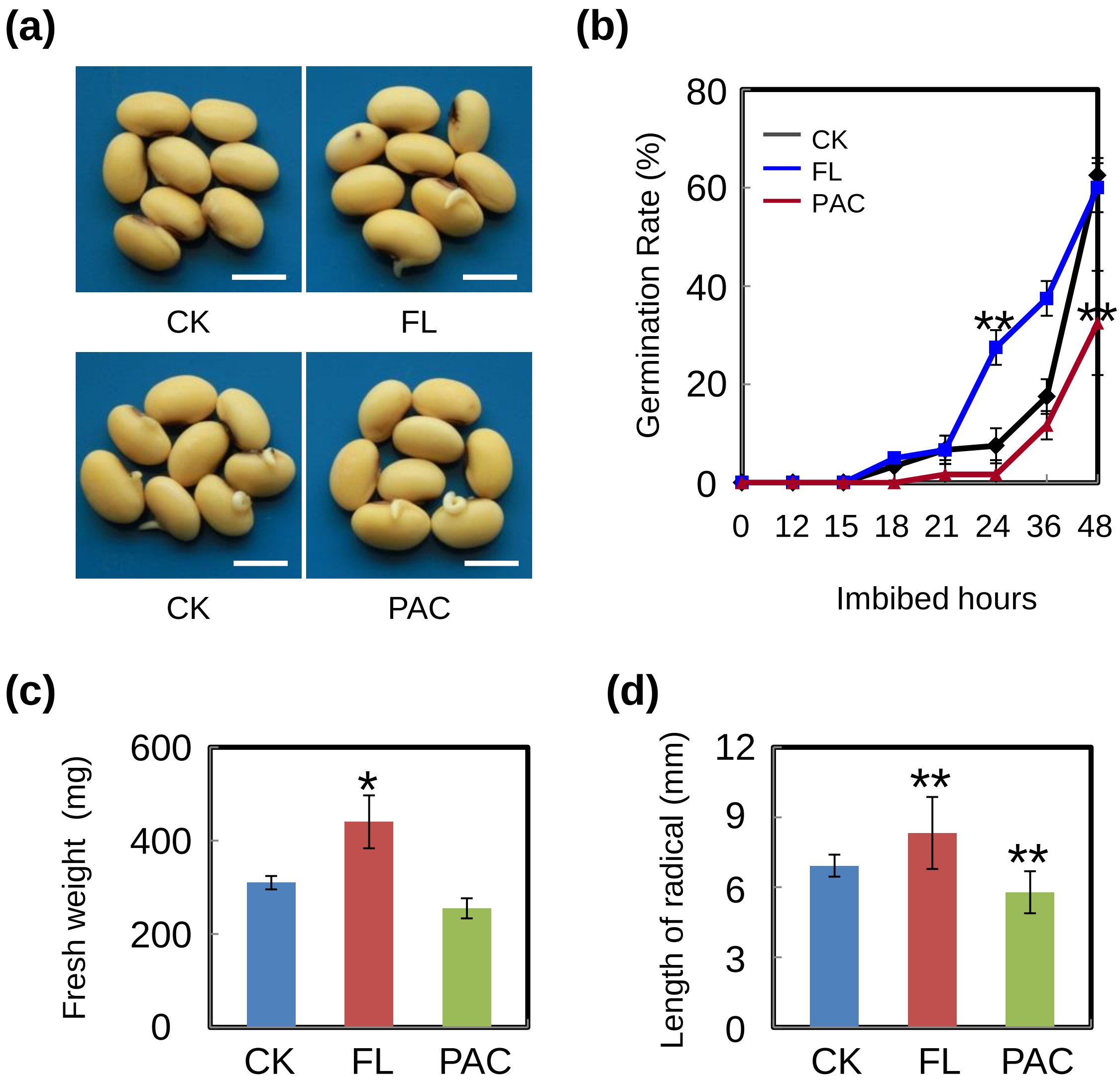


**Supplementary Fig. S5.** FL and PAC affect the germination of soybean seeds. (a) and (b) Soybean seeds (cultivar NN99) were incubated on two layers of filter paper in Petri dishes at 25 ° C under light (2430 lx) and treated with 100 nM FL and 10 μM PAC. Quantitative analysis of germination rates are shown in (b). The representative images (24 h after sowing) are shown in (a). Fresh weight (c) and radicle length (d) of germinated seeds were measured under FL treatment, PAC treatment and CK. The average length and fresh weight of four repeats are shown. ** Difference is significant at the 0.01 level, while * Difference is significant at the 0.05 level. Bar = 10 mm.

Supplementary Table 1. The $GA_4$ concentration after KAR treatment during soybean seed germination process.

| $GA_4$ Concentration (ng/g) | 0 h | 1 h | 3 h | 6 h |
|---|---|---|---|---|
| CK | 0.02±0.00 | 0.05±0.01 | 0.10±0.00 | 0.12±0.00 |
| KAR treatments | 0.02±0.00 | 0.07±0.01 | 0.09±0.00 | 0.11±0.00 |

**Supplementary Table 2.** Primer sequences used in this study.

| **For the qRT PCR** | |
|---|---|
| ***GmSTH7-Fw*** | GCCTCCGTCTTCTGCTCTG |
| ***GmSTH7-Rev*** | CTTGTTGGCGTGGTGAATGG |
| ***GmKUF1-Fw*** | GGATGTTACACGGTTGGA |
| ***GmKUF1-Rev*** | CCACGAGTCTTCTTCTTCC |
| ***GmAAO-Fw*** | AACTGAAGAAGACACCAACAAG |
| ***GmAAO-Rev*** | CTACGCAAGCACCACAAC |
| ***GmABI5-Fw*** | CGAGTTCCAGCACAGTCT |
| ***GmABI5-Rev*** | TGTTCTCTTCAGCGTTCCA |
| ***GmABI4-Fw*** | GAATCAACAGCAACAGCAACA |
| ***GmABI4-Rev*** | ACCGAAGAAGCATCCATAGC |
| ***GmRD29A-Fw*** | GGAAGGAAGAGCCAGTGA |
| ***GmRD29A-Rev*** | AACCAAGAGCCAACAACAC |
| ***GmGA3ox1-Fw*** | GCCTCCTCCAAGACATTCAA |
| ***GmGA3ox1-Rev*** | AGCCATCAACACCGTCAG |
| ***GmGA3ox-Fw*** | CTCGCATCTCTTCCTTCTTCC |
| ***GmGA3ox-Rev*** | AATCCAACATCAGCCACATCAG |
| ***GmKAO-Fw*** | TTGGAGGAGGAAGTAGATTGTG |
| ***GmKAO-Rev*** | TGCTCTTAGGTTGTTGTAGATGAA |
| ***GmGA3-Fw*** | AGATTGAACGCACCACACCTT |
| ***GmGA3-Rev*** | TCGCAGGAAGAAGAAGAGGATAGA |
| ***GmActin11-Fw*** | ATCTTGACTGAGCGTGGTTATTCC |
| ***GmActin11-Rev*** | GCTGGTCCTGGCTGTCTCC |
